# Associations between early postoperative pain outcome measures and late functional outcomes in patients after knee arthroplasty

**DOI:** 10.1371/journal.pone.0253147

**Published:** 2021-07-28

**Authors:** Emilija Dubljanin Raspopović, Winfried Meissner, Ruth Zaslansky, Marko Kadija, Sanja Tomanović Vujadinović, Goran Tulić

**Affiliations:** 1 Clinic for Physical Medicine and Rehabilitation, Clinical Center Serbia, Belgrade, Serbia; 2 Faculty of Medicine, University of Belgrade, Belgrade, Serbia; 3 Department of Anesthesiology and Intensive Care, Jena University Hospital, Jena, Germany; 4 Clinic for Orthopaedic Surgery and Traumatology, Clinical Center Serbia, Belgrade, Serbia; Jinling Clinical Medical College of Nanjing Medical University, CHINA

## Abstract

**Introduction/Aim:**

Early rehabilitation, return to daily life activities and function are the ultimate goals of perioperative care. It is unclear which pain-related patient-reported outcome measures (PROM) mirror treatment effects or are related with early and late functional outcomes.

**Methods:**

We examined associations between two approaches of pain management (scheduled vs ‘on demand’) and PROMs on post-operative days one and five (POD1, 5) with function on POD5 and 3 months after surgery in patients undergoing Total Knee Arthroplasty (TKA) in a single centre. The scheduled pain management consisted of pain assessment and routine administration of non-opioid drugs, and a weak opioid based on severity of pain reported by patients. The ‘on demand’ group received non-opioids and/or a weak opioid only when asking ‘on demand’ for analgesics.

**Results:**

On POD1, patients in the scheduled treatment group reported reduced severity of worst pain, less interference of pain with activities in-bed and sleep, and a higher proportion got out of bed. On POD5, these patients reported as well significantly less worst pain, spent significantly less time in severe pain, experienced less interference of pain with activities in bed, and felt less helpless. Furthermore, tests of function, extension and flexion ranges, Barthel index and 6 minutes walking test on POD5, and the Knee Injury and Osteoarthritis Outcome Score (KOOS) 3 months later were significantly better in the scheduled treatment group compared to the ‘on demand’ treatment group. Pain related PROMs assessed at POD1 and especially at POD5 are associated with better knee range of motion, better performance in activities of daily living, and faster gait speed, as well as less pain, better performance in activities of daily living, as well as higher knee-related quality of life 3 months postoperatively.

**Conclusions:**

Our study demonstrates that severe postoperative pain after TKA might have long lasting consequences, and even small improvements in treatment, although being far from optimal, are accompanied by improved outcomes.

## Introduction

Orthopaedic procedures on the extremities can result in severe postoperative pain [1]. Severe postoperative pain is related with impaired functional outcome in the early days after surgery, increased incidence of complications, and development of chronic postoperative pain in many surgeries including total knee arthroplasty (TKA). Despite many efforts to improve postoperative management, acute pain is still undertreated [2]. There are a significant number of studies investigating the impact of pain treatment on rehabilitation outcomes after TKA [3–11]. Several studies [3, 5, 6, 8–11] evaluate the impact of analgesia on short and long term functional outcomes, but they are not able to demonstrate that the benefits of postoperative analgesia are long lasting. Furthermore, pain intensity is the most commonly used patient-reported outcome measure (PROM) in the clinical routine as well as in acute pain management research. However, it is unclear if this pain measure or a different PROM mirrors differences in or is associated with functional outcome measures in the days or months after surgery. Therefore, we used an orthopaedic surgery setting with two distinctly different approaches of postoperative care to:

- investigate the impact of two different levels of postoperative pain management on pain-related PROMs and established function scores up to 3 months after TKA
- study the association between pain-related PROMs on postoperative days 1 and 5 (POD1, POD5) and functional scores obtained at POD5 and 3 months after surgery

## Material and methods

### Setting

All adult patients ≥ 18 years with knee osteoarthritis scheduled for total knee replacement who were consecutively admitted to the Clinic for Orthopaedic Surgery and Traumatology, Clinical Center Serbia in Belgrade between January 2016 and July 2016 were enrolled in an open prospective observational cohort study. This was a prospective observational study. The study was carried out in accordance with the principles of the Helsinki Declaration and approved by the **Ethics Committee** board of the **Clinical Center** of **Serbia (**Number 2017-004244-37). Patients were included in the study after they have signed a consent form, explaining that the study aims to improve pain treatment of patients after TKA in the future, and confirming that no changes were made in the standard medical care at the moment. The study was carried out over a 6 month period.

### Patients

Consecutive patients with knee osteoarthritis who underwent TKA were approached on the first day after surgery (POD1) for recruitment. Patients meeting the following inclusion criteria were asked the join the study: 1) older than 18 years of age; 2) able to communicate; (3) gave written consent.

### Surgical technique, anesthesia, postoperative analgesia and postoperative rehabilitation program

The indication criteria for TKA were based on a hospital used scoring system taking into account pain, function, radiological changes, and failed conservative treatment. Total knee arthroplasty was performed by one of four different experienced surgeons with insertion of tricompartmental prostheses using a standard medial parapatellar approach. Cruciate-substituting designs were used. Surgery was performed in a bloodless field using a femoral tournequet 300 mmHg. At the end of the surgery a compression bandage from the toes to the mid-thigh was applied. Spinal anesthesia was performed with the patient in a sitting position. Levobupivacaine 0.5% 3 ml was injected into the subarachnoid space throught the L3-4 intervertebral space via midline approach. General anesthesia was induced with midazolam 0.05mg/kg, fentanyl 3 mcg/kg, propofol 1.5–2.0 mg/kg, and cisatracurium 0.2mg/kg. Anesthesia was maintained with sevoflurane 1MAC. No local infiltration anesthesia has been used in our study group. Postoperatively, in the recovery room, all patients received Ketorolac 30 mg/day intravenously, and Tramadol 400 mg/day.

All patients followed a standardized postoperative rehabilitation program beginning on the first postoperative day. Assisted ambulation and regular exercise to restore strength and mobility of the operated knee were performed 2 times a day for 20–30 minutes.

### Pain treatment protocol

After surgery, patients were assigned to one of two wards, depending on availability of a free bed. This assessment method mirrors the clinical routine in our hospital, and it determined the pain treatment strategy that patients received. The routine treatment protocol on one ward consisted of pain assessment and routine administration of non-opioid drugs (Paracetamol 3000mg/24h, Ketorolac 60mg/24h and Metamizol 5000 mg/24h and a weak opioid (Tramadol 400 mg/24h) based on severity of pain reported by patients (= scheduled pain control group). The WHO stepwise approach to the use of analgesics depending on pain severity was applied. Pain was assessed at least once per shift. On the second ward, patients received non-opioids (Paracetamol, Ketorolac, Ketoprofen, Metamizol) and / or a weak opioid (Tramadol) only when they experienced pain, and after asking the attending physician or nurse for analgesics. Drugs were given according to the pain score. Pain was not routinely assessed (= ‘on demand’ pain control group). Both regimens were applied on all days from POD1 to POD5. The medications were administered intravenously. Patient controlled analgesia or co-analgesics were not given to patients on either ward.

### Tools for assessing patients

On POD1, assessment included *demographic and clinical data* comprising variables such as gender, age (year of birth), weight and height, administration of opioids before admission and analgesics perioperatively, and type of anesthesia. The assessment was carried out in patients who were at least 6 hours in the ward. Preoperative functional status was assessed with the Knee Injury and Osteoarthritis Outcome Score (KOOS) [12]. The KOOS questionnaire asks patients to assess severity of pain in the knee, function and associated problems during the week before the assessment. It consists of 5 subscales; pain, other symptoms, activities of daily living (ADL), function in sport and recreation (sport/recreation) and knee related quality of life (QOL). Standardized answer options are given (5 Likert boxes) and each question is assigned a score from 0 to 4. A normalized score (100 indicating no symptoms and 0 indicating extreme symptoms) is calculated for each subscale. On POD1 and 5 patients filled in the validated *International Pain Outcomes* Questionnaire (IPO-Q) [13]. The IPO-Q was developed to ensure collecting patient-reported pain outcomes as well as clinical data in a highly standardized procedure as part of PAIN OUT (Improvement in postoperative PAIN OUTcome) project. This project is an international quality improvement and registry aimed to improve clinical care of patients with postoperative pain, in developed as well as in developing countries. The IPO-Q evaluates the following domains: severity of pain and relief from treatments; interference of pain with physical activities in and out of bed; negative affect due to pain: anxiety and helplessness; adverse effects (AE): nausea, fatigue, dizziness, itch; perception of care: wish for more pain treatment, satisfaction with pain treatment, participation in decisions about pain treatment and receipt of information about treatment. Most items were scored using an 11-point numerical rating scale (0 = null, 10 = worst possible); four questions required dichotomous yes/no replies and two a percentage scale (0–100%). Patients related all questions to the time since surgery. The data was collected by one surveyor who underwent training before he approached patients. To reduce interviewer bias, patients completed the questionnaire independently with no assistance from family or staff. However, if a patient requested help, the surveyor could assist.

Functional assessment was carried out on POD5, consisting of knee range of motion, Barthel index and 6 minute walking test) [14] and at 3 months after surgery, with the KOOS questionnaire assessing problems during the previous week. The assessment 3 months after surgery was carried out at the patient’s follow-up visit.

In this study, we aimed to answer the following questions:

Is there an impact of two different levels of postoperative pain management on PROMs, and functional scores up to 3 months after TKA, and are PROMs obtained on POD 1 and 5 associated with functional outcomes 5 days and 3 months after surgery.

### Statistics

Continuous variables are presented as mean values with standard deviation, while categorical values are summarized as absolute frequencies and percentages. Bivariate analyses were conducted to compare patient characteristics in independent groups via Students’ T test for continuous variables, and Chi squared statistics for categorical variables. Pearson correlation coefficients assess the strength and direction of the linear relationships between pairs of variables. A p value < .05 was considered statistically significant. All analyses were performed using SPSS 22.0.

According to the post-hoc computing for the chosen error of the first type 0.05, effect size of 0.8, and sample sizes n1 = 28 and n2 = 32 for the main outcome („worst pain“) the study power was 85.99%. This calculation was done in GPower 3.1.

## Results and discussion

### Baseline characteristics of the study group

Sixty patients were recruited to the study. The ‘‘on demand’ group consisted of 28 patients, of which 46% were female, with a mean age (± SD) 66.4± 6.9. Thirty-two patients were included in the scheduled pain control group, of which 50% were female, with a mean (± SD) age of 64.4 ± 9.8 years. The groups did not differ with regards to gender and age. The vast majority of patients in both groups suffered from chronic pain (pain lasting ≥3 months) in the knee. No differences were observed with respect to the incidence of chronic pain, nor its intensity between the groups. None of the patients received opioids before admission to hospital. Patients in both groups did not differ with respect to their preoperative functional status assessed with the KOOS score, nor time on the waiting list (Table 1).

**Table 1:** Patient demographics and pre-operative characteristics.

	‘On demand’ pain control (*N* = 28), n (%)	Scheduled pain control (*N* = 32), n (%)	P =
Age (years+/-SD)^a^	64.4 ± 9.8	66.4± 6.9	0.188
Gender^b^			
Female	15 (53.6)	16 (50.0)	0.685
Male	13 (46.4)	16 (50.0)	
BMI (kg/m^2^)^a^	28.5 ± 5.1	30.4 ± 6.1	0.176
Chronic pain for at least three months before admission ^b^			
Yes	26 (92.9%)	27 (84.4%)	
No	2 (7.1%)	5 (15.6%)	0.365
Intensity of chronic pain^a^	6.60+/-2.7	6.5+/-3.1	0.195
KOOS^a^	39.0+/-15.9	32.1+/-11.7	0.133
Time on waiting list (months)^a^	30.1+/-4.1	29.6+/-3.6	0.632

^a^The values are given as the mean with the standard deviation (mean ± SD)

^b^The dichotmous values (yes/no) are given as the number of patients with the percentage in parentheses.

In the scheduled treatment group, 14 (37.0%) of patients were operated under general anesthesia (GA) and 28 (67.0%) were operated under regional anesthesia (RA). In the ‘on demand’ group, 15 (53.6%) underwent GA and 14 (46.4%) had RA. There was no difference between the two groups with respect to the proportion of GA vs. RA.

#### Postoperative analgesic consumption

In the scheduled pain control group, all 32 patients received non-opioid analgesics on POD1, in the ‘on demand’ pain control group it was 14 (50%) patients. An opioid, in the form of tramadol, was administered to 17 patients (53.1%) in the scheduled pain control group, and to 8 (28.6%) patients in the ‘on demand’ control group (p<0.01) (see Table 2).

**Table 2:** Anagesics administered on the first day after surgery in the two groups.

	‘On demand’ pain control		Scheduled pain control	
	N patients (%)	Dose mg (mean ± SD)	N patients (%)	Dose mg (mean ± SD)
**Non-opioids**				
Paracetamol	5 (18)	1888.88 ± 333	9 (50)	3000 ± 0
Ketoprofen	7 (22)	225 ± 0		
Ketorolac	14 (50.0)	36 ± 0	15 (52)	60 ± 0
Metamizol	7 (22)	3542 ± 13	12 (41)	5000 ± 0
**Opioids**				
Tramadol	8 (29)	400 ± 0	17 (53)	400 ± 0

### Patient reported outcome measures and functional outcome

PROM measured at POD1 and POD5 differed significantly between the two groups. Patients in the scheduled pain control group reported significantly less worst pain, less interference of pain with activities in-bed, and felt less helpless. Additionally, on POD1, a higher proportion of these patients got out of bed, pain interfered less with sleep, they perceived more pain relief, were more satisfied with their pain treatment, reported that they received more information about possibilities of pain treatment, and fewer wished for more analgesics. On POD5, these patients reported spending significantly less time in severe pain (see Table 3).

**Table 3:** Patient reported outcomes on POD1 and POD5 in the two groups.

	POD1			POD5		
	Scheduled pain control	‘On demand’ pain control	P =	Scheduled pain control	‘On demand’ pain control	P =
Worst pain-a	6.0±3.1	7.1±2.3	.013	3.8±2.1	5.8±3.1	.005
Least paint^a^	2.1±1.8	3.1±2.5	.057	1.4±2.2	2.2±2.2	.192
Percentage of time in severe pain^a^	35.5±25.6	42.9±27.2	.284	15.5±15.3	33.3±26.4	.003
Pain interfering with activities in bed^a^	5.1±2.8	6.6±2.7	.040	2.9±2.4	4.5±2.6	.019
Pain interfering with taking a deep breath^a^	0.8±2.1	1.1±2.2	.590	0.1±.4	0.5±1.5	.179
Pain interfering with sleep^a^	3.5±3.5	5.3±3.4	.042	1.6±2.4	2.7±3.6	.214
Out of bed since surgery						
Yes	15 (50%)	5 (26%)	.005	n.a.	n.a.	
Pain interfering with activities out of bed^a^	4.7±3.1	n.a.		n.a.	n.a.	
Pain caused anxiety^a^	3.4±3.5	4.6±3.0	.138	1.7±2.9	4.1±3.3	.128
Pain caused helplessness	3.0±3.4	5.3±3.5	.013	1.1±2.4	3.6±3.3	.014
Nausea^a^	2.3±3.6	2.4±3.3	.970	1.0±2.2	0.7±1.4	.350
Drowsiness^a^	2.8±3.4	2.2±2.8	.517	1.9±3.0	1.5±2.6	.292
Itch^a^	0.0±0.0	0.2±0.6	.122	0.0±.0.0	0.2±.9	.009
Dizziness^a^	1.2±2.9	0.6±1.1	.308	3.9±16.7	0.6±1.6	.053
Percentage of pain relief^a^	63.8±25.7	40.4±25.6	.001	63.4±30.9	54.5±26.5	.163
Wish for more pain treatment						
Yes	9 (31%)	16 (52%)	.106	5 (17%)	8 (27%)	.383
Received information about pain treatment						
Yes	17 (59%)	6 (19%)	.002			
Participation in decisions regarding pain treatment^a^	7.1±2.8	6.0±3.3	.202	7.8±2.7	6.4±2.4	.894
Satisfied with the results of pain treatment ^a^	8.8±1.9	7.2±2.7	.018	8.4±2.7	8.4±1.9	.195

^a^The values are given as the mean with the standard deviation (mean ± SD)

^b^The dichotomous values (yes/no) are given as the number of patients with the percentage in parentheses. n.a. = not applicable.

On POD5, all observed functional outcome measures, knee extension deficit and flexion, Barthel score, as well as 6 minute walking test were significantly better in the scheduled pain control group. The differences between the two treatment groups regarding early functional outcome were most pronounced with respect to ambulation. Patients in the group with scheduled pain control group had a highly significant faster gait speed on POD5 as measured with the 6 minute walking test (see Table 4).

**Table 4:** Functional outcomes on POD5.

	Scheduled pain control mean ± SD	‘On demand’ pain control mean ± SD	P
Extension ^a^	-10.4±6.6	-14.2±6.3	.025
Flexion^a^	72.2±20.4	56.8±20.1	.005
Barthel index^a^	82.2±12.7	67.7±19.5	.000
6 minute test^a^	142.5±50.8	87.7±60.1	.000

^a^The values are given as the mean with the standard deviation (mean ± SD)

At 3 months postoperatively, the KOOS subscales for pain, activities of daily living and quality of life were significantly better in the scheduled pain group (see Table 5).

**Table 5:** KOOS subscales 3 months after surgery.

	Scheduled pain control	‘On demand’ pain control	P =
Symptoms^a^	64.3±6.2	64.3±15.8	.996
Pain^a^	80.3±23.4	87.0±11.2	.037
Activity of Daily Living^a^	86.9±14.2	70.1±20.7	.001
Sports^a^	23.6±25.5	16.0±29.4	.308
Quality of life^a^	73.7±24.7	46.1±22.7	.000

^a^The values are given as the mean with the standard deviation (mean ± SD)

Our results revealed weak but statistically significant associations between pain related PROMs on POD1 and functional outcome parameters on POD5 and 3 months postoperatively. Severity of ‘worst pain’, “proportion of patients out of bed” and “percentage of pain relief” on POD1 correlated with the Barthel score on POD5. “Least pain” on POD1, as well as “feeling of helplessness”, “anxiety”, and “percentage of pain relief” correlated with the degree of knee flexion on POD5. Percentage of pain relief and participation in decisions regarding pain treatment correlated significantly with the 6 minute walking test at PO5 (see Table 6).

**Table 6:** Pearson correlation coefficients r between PROMS on POD1 and functional results on POD5 and 3 months after surgery (* p < 0.05).

POD1	POD5				3 months postoperatively			
	Barthel score	Flexion	Extension	Six minute test	Symptoms	Pain	ADL	Qol
Worst pain	-.27 (.038)							
Least pain		.29 (.025)						
Time in severe pain						-.27 (.048)		
Interference with in-bed activities							-.28 (.037)	
Feeling of Anxiety		-.28 (.025)						
Feeling of helplessness		-.34 (.009)						
Percentage of pain relief	.39 (.002)	.34 (.011)		.25 (.044)		.31 (.020)		.41 (.002)
Participation in treatment decisions				.31 (.002)				
Satisfaction with pain treatment							.31 (.023)	

The pain intensity sub-score of KOOS at 3 months postoperatively correlated with the frequency of severe pain and percentage of pain relief on POD1. The quality of life sub-score 3 months postoperatively was also related to the percentage of pain relief assessed on POD1, while independence in performing ADLs 3 months postoperatively correlated with pain interference with in-bed activities and satisfaction with pain treatment on POD1 (see Table 6).

Significant relationships were also observed between pain related PROMs on POD5 and functional outcome measures assessed on POD5 and 3 months postoperatively. Patients who were mobilized out of bed in the last 24 hours, and felt less helpless on POD5 performed better regarding the Barthel score on POD5. Furthermore, patients who had lower scores for worst and least pain, lower frequency of severe pain, and who were more involved in pain decisions on POD5 achieved higher range of motion in terms of flexion on POD5. Pain interference with activities out of bed was related with a higher extension deficit on POD5, while patients who felt anxious on POD5 performed worse on the 6 minute test on POD5 (see Table 7).

**Table 7:** Pearson correlation coefficients r between pain, functional interference and perception of care on POD5 and functional outcomes at POD5 and 3 months after surgery (* p < 0.05).

POD5					3 months postop.			
POD5	Barthel index	Flexion	Extension	Six min test	Symptoms	Pain	ADL	Qol
Worst pain		-.26 (.046)				-.38 (.004)	-.35 (.009)	-.53 (.000)
Least pain		-.30 (.023)				-.30 (.016)		
Time in severe pain		-.40 (.026)				-.33 (.015)	-.30 (.024)	-.34 (.010)
interference with in-bed activities						-.33 (.013)	-.38 (.004)	-.36 (.007)
Interference with sleeping						-.32 (.019)	-.31 (.010)	
Interference with activities out of bed			.41 (.003)			.32 (.032)	-.29 (.047)	-.37 (.011)
Feeling of Anxiety				-.28 (.036)				-.37 (.005)
Feeling of Helplessness	-.27 (.042)							
Percentage of pain relief					.32 (.018)	.28 (.043)		
Participation in treatment decisions		.36 (.006)				.27 (.049)	.31 (.023)	.33 (.015)
Satisfaction with pain treatment								

Moreover, a number of IPO-Q items on POD5 correlated significantly with functional outcome 3 months later, specifically worst pain, time in severe pain, interference with activities and participation in treatment (see Table 7).

## Discussion

This study investigated the impact of two different levels of postoperative pain management on pain-related PROMs and established function scores up to 3 months after TKA and the association between pain-related PROMS and functional recovery scores at different time points, up to 3 months after surgery in patients undergoing TKA. Pain management was not optimal on either ward in that patients reported moderate to severe pain, they spent on average 36–43% of the first postoperative day in severe pain and many would have wanted more treatment for pain than they received. However, administration of higher doses of analgesics in the scheduled treatment group were associated with reduced pain-related PROMs and improved early functional outcome during the first days after surgery. This treatment regimen was also related with positive effects on pain and function lasting for at least 3 months postoperatively. A number of pain-related PROMs obtained early after surgery were associated with functional outcome measures at POD5 as well with the KOOS score 3 months postoperatively. Generally, several understandings emerge from the correlation analysis. First, the PROM "percentage of pain relief" measured on POD1 correlated with the largest number of outcome measures on POD5 and 3 months postoperatively. Its predictive value could, thus, be further investigated in future multivariate regression analyses. Second, PROMs assessed on POD5 had higher predictive values with short and long term rehabilitation outcomes as compared to PROMs obtained on POD1. Therefore, a critical evaluation of PROMs and function several days after surgery might identify patients at risk for worsened functional outcome later on. Third, the PROMs reported on POD5 that showed the largest number of correlations with observed functional outcome measures were "worst pain", "time in severe pain", and "participation in decisions about pain treatment options".

Our study demonstrated that decreased pain management immediately after TKA might have long lasting consequences, and even small improvements in treatment, although being far from optimal, are accompanied by improved outcomes. Therefore, effective pain management is of utmost importance. Our findings reinforce the need for providing adequate pain management. There is considerable evidence from literature describing the benefits of effective analgesia on PROMs, better knee function, and better walking ability in the early postoperative period [6–9, 11]. In studies investigating outcome after 6 weeks [3, 4, 6, 9], positive effect of improved analgesia was confirmed with respect to improved pain profile [3, 6], greater range of motion in the operated knee [6], and better early postoperative activity measures (2 minute walking test and walking time) [4].

There is an increasing debate as to the role of PROMs in the area of acute pain management. Pain intensity alone may not to be an ideal measure of quality of care, and it is unclear if and to what extent it mirrors functional capabilities. Van Boeckel at al. recently showed that pain intensity did not correlate well with patient’s opinion on acceptability of pain, nor with nurses’ observation of patient’s performance of necessary activities [15]. However, Chapman et al. [16] using PAIN OUT data showed that worst pain scores were moderately to strongly associated with functional PROMS on the first post-operative day in orthopaedic patients. Our current findings show that in the days after surgery PROMS other than worst pain can be used to evaluate recovery. This may be in line with findings by Fletcher et al. [17] who showed that the duration spent in severe pain on the first post operative day was the PROM best predicting chronic pain in patients 12 months after orthopedic surgery.

Our study is associated with several limitations. This was not a randomized trial. We cannot claim that selection bias was eliminated though the principle difference between the two groups was that patients were allocated to one of the two wards, and this depended on availability of a bed. We only studied a small number of patients in a single center with lack of health care personnel, including potential surveyors. In our study only one surveyor collected patient-reported outcome data as well as clinical data in a highly standardized way using the IPO-Q questionnaire and inputted the data into the webbed data entry mask, which is a very time consuming process. Therefore, we were forced to limit the study investigation period to 6 months, and consequently include a relatively small number of patients. Larger sample sizes in other studies were the same IPO-Q questionnaire was used were obtained by gathering datasets from multiple hospitals in different countries [17]. Furthemore, other authors published their results using the KOOS scale as an outcome measure on patients undergoing TKA using a small study group as well [18, 19]. Although, statistically significant associations were found between PROMs obtained at POD1 and 5 and functional outcome measures, the correlations are weak. Clinical relevance should be evaluated, but this would require larger patient groups. Additionaly, data included patient operated under general and spinal anesthesia without differentiation. Lastly, we did not correct for multiple comparisons as we consider that this as a hypothesis generating study. The uncorrected correlations point towards relationships which might be interesting for investigation in future studies with larger patient populations and/or different settings.

Our study is novel in that it was carried out in a low-resource country, and mirrors reality of management in a low resource setting, with limited education and awareness regarding pain therapy. It might be considered to be ethically questionable to expose patients to a setting with known suboptimal care patients. However, patients would not have been treated differentially without this study, and the results would not have been reported. Therefore, we hope that findings such as ours will encourage other care givers working in similar conditions to evaluate outcomes of patients they care for. Our study also illustrates that standardized data collection offers an indispensable tool for bringing to light information about PROMS and approaches for managing pain.

## Conclusions

Although quality of pain management in patients evaluated in this study is different from standards reported in high resource countries, we showed that even slightly increased pain management translates in reduced severity of pain and in improved functional outcomes. Availability of information about deficits in care can serve as a catalyst for providers to bring about change. Moreover, early PROMs can be used to detect patients at increased risk for delayed functional recovery after TKA. These conclusions should motivate care givers to improve the perioperative pain management they provide to their patients after TKA.

## Supporting information

S1 Raw data (XLSX)
